# Understanding the interplay between COVID-19 and diabetes: insights for the post-pandemic era

**DOI:** 10.3389/fendo.2025.1599969

**Published:** 2025-05-21

**Authors:** Xuefei Zhao, Linlin Jiang, Wenjie Sun, Shanshan Tang, Xiaomin Kang, Qing Gao, Zehua Li, Xuedong An, Fengmei Lian

**Affiliations:** ^1^ Guang’anmen Hospital, China Academy of Chinese Medical Sciences, Beijing, China; ^2^ Beijing University of Chinese Medicine, Beijing, China; ^3^ College of Traditional Chinese Medicine, Changchun University of Traditional Chinese Medicine, Changchun, China

**Keywords:** COVID-19, SARS-CoV-2, diabetes, glucose metabolism, pancreatic β-cells, virus induced diabetes

## Abstract

The global pandemic caused by the SARS-CoV-2 virus has had a profound impact on the onset, progression, and management of diabetes, posing significant challenges to healthcare systems worldwide. This review elucidates the multifaceted impact of SARS-CoV-2 on diabetes mellitus, emphasizing the increased complexity of glycemic management in patients with SARS-CoV-2 infection following viral infection in the postpandemic era. In this study, we examined the diverse effects of the SARS-CoV-2 virus on individuals with diabetes. These effects included an elevated risk of morbidity, erratic fluctuations in blood glucose levels, the emergence of complications associated with diabetes, and the emergence of challenges related to self-management of the disease. From a mechanistic perspective, we investigated the following factors: SARS-CoV-2-mediated direct damage to islet beta cells, dysregulation of the RAAS system, impairment of islet function by oxidative stress, and the effects of the integrated stress response, stress response, and reduced adiponectin levels on insulin utilization efficiency and glucose metabolism. Furthermore, viral effects extend to diabetic complications and cardiovascular risk factors, such as coagulation abnormalities, hypertension, and lipid metabolism. This results in an exacerbation of the development of diabetic complications. This review highlights the urgent need for refined management strategies for patients with diabetes during the pandemic and in the later stages of COVID-19. Additionally, there is a need for integrated management strategies to mitigate the impact of COVID-19 on the long-term outcomes of patients with diabetes.

## 1 Introduction

Although the acute threat of the coronavirus disease 2019 (COVID-19) pandemic has diminished, its shadow lingers in the form of post-acute sequelae (PASC), posing unprecedented challenges to chronic disease management—particularly for diabetes mellitus (DM), a metabolic disorder characterized by chronic hyperglycemia and multisystem comorbidities (1). DM has a significant impact on the quality of life and overall health of patients and places a burden on national healthcare resources. The bidirectional relationship between severe acute respiratory syndrome coronavirus 2 (SARS-CoV-2) and diabetes remains alarming: while diabetic patients face elevated risks of severe COVID-19 outcomes (2), emerging evidence implicates the virus as a catalyst for new-onset diabetes and glycemic destabilization (3). This includes an increased risk of morbidity, fluctuating blood glucose levels, an increased likelihood of diabetic complications, and self-management challenges, which will be described in greater detail later. This review delves into several key aspects of its mechanism, including the direct effects of the virus on pancreatic beta cells, as well as the broader metabolic dysregulation associated with infection. A meta-analysis of 11 retrospective cohorts spanning 47.1 million subjects globally underscores this peril, revealing a 64% increased risk of incident diabetes post-COVID-19 (4). As global healthcare systems are now coping with the long-term complexities of the aftermath of COVID-19, there is a pressing need to investigate the mechanisms through which the virus affects DM from various perspectives and to develop a comprehensive management strategy to address the distinctive challenges encountered by individuals with DM. The objective of this review is to provide a comprehensive examination of the complex interrelationship between SARS-CoV-2 and DM, emphasizing the multifaceted influence of viral infection on glycemic control and DM management.

## 2 Impact of COVID-19 on the development and clinical management of DM

### 2.1 COVID-19 increased the incidence of DM

#### 2.1.1 The impact of SARS-CoV-2 infection on the incidence of DM

There is a bidirectional relationship between SARS-CoV-2 infection and DM. On the one hand, diabetic patients tend to develop more severe clinical symptoms after contracting COVID-19. Conversely, inadequate glycemic control in individuals with newly diagnosed DM and in those with preexisting DM has also been reported in SARS-CoV-2-infected individuals, which may be one of the complications of COVID-19 (5, 6). The varying degrees of glucose intolerance that patients develop after COVID-19 are referred to as ‘long COVID’ or ‘post-COVID syndrome’ (7). Statistical analysis indicated that the risk of developing DM may be increased by 60% in individuals infected with SARS-CoV-2. However, the incidence of DM is greater than that of respiratory infections caused by other viruses (8). The glycemic abnormalities caused by COVID-19 include both type 1 DM (T1DM) and type 2 DM (T2DM). Consequently, it is imperative to prioritize the investigation of T1DM in pediatric populations (9). A meta-analysis of 11 retrospective cohorts (47.1 million participants) revealed that patients with COVID-19 had a 64% greater risk of developing DM (RR = 1.64, 95% CI: 1.51–1.79), including both T1DM and T2DM, than non-COVID-19 controls did (4). In addition, a prospective cohort study of 181,280 infected individuals over 352 days revealed an increased risk (HR 1.40, 95% CI 1.36 - 1.44) and excess burden (13.46, 95% CI 12.11 - 14.84, per 1000 people at 12 months) of DM in COVID-19 patients compared with controls (10).

COVID-19 is characterized by the overproduction of inflammatory factors such as interleukins (ILs), interferons (IFNs), chemokines and tumor necrosis factors (TNFs). Viral infection leads to overactivation of immune cells and the production of proinflammatory cytokines, known as a cytokine storm, which further leads to severe COVID-19 symptoms and patient death (11). In addition, this inflammatory storm damages organs throughout the body (including major glucose-metabolizing organs) and increases insulin resistance. The pancreas is an important target for SARS-CoV-2 because angiotensin-converting enzyme 2 (ACE2) is expressed in both exocrine glands and pancreatic islets (12), and inflammation of pancreatic beta cells in COVID-19 patients also contributes to new-onset DM (13). These possible reasons for the increased incidence of DM after SARS-CoV-2 infection will be discussed in more detail later.

#### 2.1.2 The impact of COVID-19 treatment on the incidence of DM

In addition to the damage to glucose metabolism caused by viral infection, treatment for COVID-19 can also cause blood glucose abnormalities. Diabetic drugs such as corticosteroids, which are often used in patients with severe COVID-19, are major causes of DM (14). Dexamethasone reduces mortality and the length of hospital stay in patients with severe COVID-19 (15), but it increases blood glucose levels, making it not recommended for patients with mild COVID-19 and DM (16). In addition to dexamethasone, some studies have suggested that the antiviral drug *Remdesivir* also leads to high blood glucose levels, which should be taken into account when caring for patients with both DM and COVID-19 (17). Lifestyle changes during the COVID-19 pandemic may also contribute to DM. During the COVID-19 epidemic, the population adopted unhealthy diets and reduced physical activity as a result of widespread confinement. These lifestyle changes contribute to obesity, exacerbating insulin resistance, which may accelerate the progression to T2DM in individuals with preexisting β-cell dysfunction (18). This increase in the prevalence of obesity during the COVID-19 pandemic has also been reported in other studies (12). Notably, just 2 weeks of reduced daily physical activity in healthy young adults resulted in decreased muscle mass, increased visceral fat mass, insulin resistance and increased plasma triglyceride levels (19).

### 2.2 COVID-19 affects glucose levels in diabetic patients

COVID-19 itself is thought to trigger new-onset diabetes and hyperglycemia but also to worsen glycemic control in patients with pre-existing diabetes. COVID-19 is not uncommon in patients with established diabetes mellitus in the context of the SARS-CoV-2 pandemic (14). Poor glycemic control was observed in both T1DM and T2DM patients after SARS-CoV-2 infection, and the reasons for these two conditions were different. The former is attributed to a cytokine storm caused by the abnormal release of all kinds of hormones and various proinflammatory cytokines, and the latter may be due to the massive destruction of pancreatic beta cells by the virus (20). People with DM after COVID-19 experience both hyperglycemia and blood glucose fluctuations.

#### 2.2.1 COVID-19 causes elevated blood glucose in patients with DM

Following infection with SARS-CoV-2, the onset of hyperglycemia may precede symptoms of the disease itself (20). A study of 29 patients with T2DM reported that 12% of diabetic patients experienced frequent hyperglycemia after infection (21). This was associated with higher rates of hospitalization and mortality (22). The presence of corticosteroids, cytokine storms, damage to pancreatic beta cells, or a combination of these factors has been demonstrated to result in more severe hyperglycemia and increased ketoacidosis in individuals with DM (23). In addition, the strategies implemented by governments during the pandemic also contributed to elevated blood glucose levels. The results of a systematic review and meta-analysis demonstrated that the implementation of a lockdown due to the pandemic resulted in statistically significant increases in HbA1c, fasting glucose, and body mass index in patients with T2DM (p < 0.05) (24).

#### 2.2.2 COVID-19 causes fluctuations in blood glucose in patients with DM

In the management of blood glucose in individuals with DM, preventing hyperglycemia and maintaining a stable blood glucose level are equally important. Hypoglycemia is a common complication of DM and is more likely to result in adverse clinical outcomes, including mortality, than is hyperglycemia. Typically, hypoglycemia is attributed to substance abuse (especially insulin use) and poor dietary management. However, this effect appears to be intensified in critically ill patients in the intensive care unit (ICU) (25). The majority of available evidence indicates that the severity and mortality of SARS-CoV-2 infection are greater in individuals with DM, particularly when accompanied by fluctuations in blood glucose levels (26). Notably, some studies have reported a correlation between insulin use and mortality in hospitalized patients (27). Hypoglycemia incidence is elevated in hospitalized COVID-19 patients with diabetes, likely due to erratic oral intake, altered drug metabolism, and cytokine-mediated insulin resistance (28).

### 2.3 The impact of COVID-19 on the development of diabetic complications

Following a 12-week period during which the patient has been infected with SARS-CoV-2, a number of clinical symptoms may still be observed, including fatigue, dyspnea, myalgia, weakness, headache and cognitive retardation. These symptoms are collectively known as post-COVID-19 syndrome. The incidence of microvascular dysfunction and organ damage is increased in individuals with DM following a diagnosis of SARS-CoV-2 infection. Combined with hospitalization, protein deficiency, and corticosteroid therapy, there is also a loss of muscle mass and increased fatigue, especially in the elderly and women (29). Furthermore, the incidence of complications has been shown to be elevated in individuals with DM following a diagnosis of SARS-CoV-2 infection (30).

#### 2.3.1 Cardiovascular and cerebrovascular complications

The SARS-CoV-2 virus can accelerate the development of T2DM, which in turn can lead to long-term complications of T2DM (31). SARS-CoV-2 infection has been demonstrated to cause damage to vascular endothelial cells, thus increasing the risk of cardiovascular and cerebrovascular disease among diabetic patients. Compared with cardiovascular and cerebrovascular complications caused by DM alone, these complications are more likely to occur with the additional presence of SARS-CoV-2 infection (12). In patients with DM and concomitant infection with the SARS-CoV-2 virus, hyperglycemia, hypoxia and proinflammatory environments lead to vascular endothelial dysfunction and thrombosis, thereby increasing the risk of cardiovascular and cerebrovascular complications (32). Patients with basal metabolic dysfunction, such as T2DM and obesity, are at increased risk of developing complications from SARS-CoV-2 infection, including multiple organ dysfunction and cellular energy deprivation secondary to immune response disorders (33). At present, the long-term effects of COVID-19 on cardiovascular and cerebrovascular complications remain unclear because of the absence of high-quality clinical studies.

#### 2.3.2 Chronic complications of DM

The chronic complications of DM include diabetic retinopathy (DR), diabetic nephropathy, diabetic peripheral neuropathy (DPN) and diabetic foot. While case reports suggest potential associations between SARS-CoV-2 infection and worsening microvascular complications, robust evidence linking direct viral mechanisms to retinopathy, nephropathy, or neuropathy remains limited. The observed increases in complications may reflect pandemic-related disruptions in care, psychological stress, or indirect metabolic dysregulation rather than direct viral effects. The monitoring and treatment of DR during the current pandemic are facing significant challenges. During the pandemic, there has been a notable decline in the administration of intravitreal injections for DR worldwide, with reductions ranging from approximately 30% to nearly 100% (34). To date, no research has been conducted to confirm the effect of the SARS-CoV-2 virus on the development of DR; however, a number of case reports have mentioned this possibility (35, 36). A five-year retrospective study revealed a notable surge in the incidence and severity of DR following the onset of the pandemic. The authors posit that this may be related to the psychological impact of the pandemic rather than the direct effects of the virus itself (37). Furthermore, studies have reported the effects of the SARS-CoV-2 virus on DPN (38), with evidence suggesting that vaccines may contribute to this phenomenon (39). Diabetic nephropathy (40) and diabetic foot (41) have also been mentioned, but these studies lack supporting clinical evidence.

#### 2.3.3 Acute complications of DM

Individuals with diabetes infected with the SARS-CoV-2 are more susceptible to developing severe acute complications of DMI, including diabetic ketoacidosis and hyperosmolar hyperglycemia (42). The entry receptor of SARS-CoV-2, ACE2, is expressed in several extrapulmonary tissues and may cause hyperglycemia and ketosis (43). Metformin and sodium–glucose transport protein 2 inhibitors (SGLT2is) may be discontinued in patients with severe COVID-19 because of their association with lactic acidosis and ketoacidosis (20). A study of 658 hospitalized patients with confirmed SARS-CoV-2 infection demonstrated the occurrence of ketosis in 42 patients. Among these patients, three presented with diabetic ketoacidosis, and four died (44).

### 2.4 COVID-19 affects self-management in diabetic patients

The results of a systemic review show that DM patients who maintained or increased physical activity during the COVID-19 pandemic had lower plasma glucose and HbA1c levels. Some authors suggested that COVID-19 promoted deleterious metabolic adjustments in DM patients and that physical activity followed by exercise would have beneficial effects on insulin sensitivity and lipid metabolism homeostasis (45). The implementation of lockdown measures during an epidemic has adverse effects on the medical treatment and exercise regimens of diabetic patients, which in turn impedes the control of blood glucose levels. During the lockdown, all individuals, including those with T2DM, are permitted only those activities deemed essential. Such a change in lifestyle, including diet, exercise, insulin adjustment, mood, stress, social relationships and work activities, can have a significant impact on both physical and mental health, potentially affecting self-management and glycemic control in individuals with DM (18). Furthermore, for those with DM complications, the ongoing pandemic may also result in delays or even the cancellation of planned treatments (34).

### 2.5 Impact of COVID-19 on clinical management decisions in diabetic patients

#### 2.5.1 Increased rates of hospitalization and risk of death

Patients with T1DM or T2DM have a markedly elevated risk of severity and mortality from SARS-CoV-2 infection, particularly those with poor glycemic control (26). Reports show that patients with DM concurrently infected with the SARS-CoV-2 virus are at an elevated risk of mortality (46). Existing data indicate that elevated blood glucose levels facilitate local viral replication in the lungs, impair the antiviral immune response, and ultimately result in severe illness in patients with COVID-19 (47). A meta-analysis involving 45,775 hospitalized COVID-19 patients revealed that the weighted prevalence of mortality in hospitalized COVID-19 patients with DM (20.0%, 95% CI: 15.0–26.0) was 82% higher than that in non-DM patients (11.0%, 95% CI: 5.0–16.0) (22).

#### 2.5.2 Drug treatments

Insulin is a key therapy for the treatment of diabetes and is used in patients suffering from severe COVID-19 accompanied by hyperglycemia (14). However, some studies have mentioned an increase in mortality in these patients to whom insulin therapy has been applied (48). A retrospective study of 3,305 cases from Wuhan, China, indicated that insulin treatment for patients with COVID-19 comorbid with T2DM was associated with a significant increase in mortality (27.2% versus 3.5%) (28). Insulin therapy has the potential to cause a hypoglycemic response. While mortality was not reduced in insulin-treated COVID-19 patients even in the absence of hypoglycemia (27). It is important to note that insulin remains the cornerstone therapy for hyperglycemic ICU patients, including those with undiagnosed diabetes mellitus, as it is effective in relieving acute hyperglycemia and ketoacidosis. The association between insulin use and mortality observed in the retrospective study may reflect confounding by indication, as COVID-19 patients to whom insulin is applied are usually critically ill patients with severe hyperglycemia or comorbidities (e.g., cardiovascular disease). Therefore, the reasons for the increased mortality rate may be complex, with increased systemic inflammation and damage to vital organs as potential causes. It is recommended that insulin use be measured and fully evaluated by clinicians.

Other oral hypoglycemic agents have different effects on diabetic patients with COVID-19, and some may be more suitable for diabetic patients with concurrent COVID-19. Current evidence indicates that drugs commonly used for the treatment of DM, including dipeptidyl peptidase 4 (DPP-4) inhibitors, glucagon-like peptide-1 receptor agonists (GLP-1RAs), SGLT-2is and metformin, may influence the progression of SARS-CoV-2 infection. Therefore, it is necessary to determine the efficacy and safety of these agents in the treatment of DM patients with SARS-CoV-2 infection (49). The administration of peroxisome proliferator-activated receptor γ (PPARγ) activators and GLP-1RA has been demonstrated to upregulate ACE2 in animal models, which may increase the risk of SARS-CoV-2 infection (50). Metformin has been proven to reduce lung damage and is therefore regarded as a promising treatment for patients with DM and COVID-19 because of its dual activity (51). DPP-4 inhibitors, such as gliptins, may have the potential to exert a positive pleiotropic effect on inflammatory diseases and could therefore be repurposed as salutary drugs against COVID-19 syndrome, even in nondiabetic subjects (52). SGLT2i has also been suggested to be beneficial for COVID-19 outcomes; however, these potentially beneficial agents remain controversial, with an insufficient number of clinical trials (53). In addition, herbs such as curcumin and propolis may have adjunctive antiviral, anti-inflammatory, insulin-resistance-reducing, and immunomodulatory effects and show promise in assisting the management of COVID-19 and DM comorbidity (54). Specific herbs and traditional formulations have potential benefits in controlling symptoms, regulating blood glucose levels, and improving immune function, but there is insufficient clinical evidence to support their use as stand-alone therapies (55).

### 2.6 Evaluation and management

During the COVID-19 pandemic, the assessment and management of people with DM has been affected (56). The outbreak had an enormous impact on national health services, particularly on the diagnosis and monitoring of chronic diseases such as DM (57). In the postepidemic era, this impact is also subtly changing the management model for DM patients. During the pandemic, the pace of development of digital services began to increase exponentially. As actual doctor–patient access is limited, telemedicine offers the most convenient opportunity to communicate with and maintain the care of people with DM (58). In fact, telemedicine is the most useful and commonly used tool for maintaining contact between patients and doctors when physical distance is limited by confinement (59), and telemedicine is more appropriate for the assessment and management of chronic diseases such as DM. Nevertheless, there are still significant challenges in telemedicine, such as a fragmented approach to health technology assessment and reimbursement, a lack of eHealth education and literacy, particularly among healthcare professionals, a lack of data integration, and concerns about electronic health records, patient consent and privacy (60). However, such measures are beneficial for the management of people with DM and may prove invaluable in the event of a similar outbreak in the future. Clinical studies on the effects of COVID-19 on DM are summarized in **Table 1**.

**Table 1:** Research on COVID-19 and DM.

First Author (year)	Study	Population (control group)	Outcome	Conclusion
(10)	Cohort study	4229721 (4118441)	Risks of incident DM Antihyperglycemic use	COVID-19 increases the risks of incident DM and antihyperglycemic use
(123)	Retrospective cohort study	71730 (35865)	Incidence rate ratios (IRRs) for DM.	COVID-19 confers an increased risk for type 2 DM
(124)	Real-World Study	27292879 (24803613)	The odds of developing new-onset T1D The odds of developing DKA	COVID-19 diagnosis is associated with significantly increased risk of new-onset T1D, and the risk of developing DKA is significantly increased following COVID-19 diagnosis.
(125)	Cohort study	592003 (514656)	Deep vein thrombosis (DVT), pulmonary embolism (PE), ischemic stroke, myocardial infarction (MI), heart failure, AKI and new T2DM diagnosis	Risk of cardiometabolic and pulmonary adverse outcomes is markedly raised following discharge from hospitalization with COVID-19 compared to the general population.
(126)	Retrospective cohort study	717070 (468173)	The presence of persistent and new sequelae at 21 or more days after a diagnosis of COVID-19	The results confirm an excess risk for persistent and new sequelae in adults aged ≥65 years after acute infection with SARS-CoV-2.
(127)	Cohort study	994722 (394667)	The risk for new-onset T2DM within 180 days	The study found that COVID-19 was associated with an increased risk for new-onset T2DM compared to influenza in a multi-institutional research network.
(128)	Retrospective cohort study	520332 (439439)	Incident DM	These data suggest an increased risk for DM among persons aged <18 years with COVID-19
(129)	Retrospective cohort study	95560 (47780)	Rates of hospital readmission All-cause mortality Diagnoses of respiratory, cardiovascular, metabolic, kidney, and liver diseases	Individuals discharged from hospital after COVID-19 had increased rates of multiorgan dysfunction compared with the expected risk in the general population.
(130)	Retrospective cohort study	1165505 (898919)	Clinical sequelae	The results indicate the excess risk of developing new clinical sequelae after the acute phase of SARS-CoV-2 infection, including specific types of sequelae less commonly seen in other viral illnesses.

## 3 Mechanisms of action of SARS-CoV-2 in the etiology of DM

### 3.1 Impairment of pancreatic islet function leads to insufficient insulin production

#### 3.1.1 Direct damage to pancreatic beta cells caused by SARS-CoV-2, with both acute and sustained effects

New-onset hyperglycemia after SARS-CoV-2 infection may be attributed to the ability of the virus to successfully invade beta cells, abduct their intracellular mechanisms and disrupt their insulin production. SARS-CoV-2 gains entry into beta cells via viral receptors, as well as subsequent damage due to direct acute viral injury or the long-term persistence of uncleared virus. In both instances, SARS-CoV-2 directly induces β-cell dysfunction and death (61, 62).

*In vitro* studies have demonstrated a notable reduction in the prevalence of SARS-CoV-2 in other pancreatic α and δ cells, as well as in endothelial cells. Conversely, human pancreatic beta cells exhibit heightened vulnerability to SARS-CoV-2 (63). Trajectory analysis demonstrated that SARS-CoV-2 induces eIF2 pathway-mediated beta cell transdifferentiation, resulting in decreased insulin gene expression in beta cells. Concurrently, glucagon and other alpha cells and acinar cell markers are upregulated in beta cells (11, 64). Autopsy samples from COVID-19 patients demonstrated a reduction in insulin expression and the abnormal presence of trypsin/insulin double-positive cells, confirming that SARS-CoV-2 induces such transdifferentiation of beta cells (11). SARS-CoV-2 has been demonstrated to directly induce β-cell killing. The SARS-CoV-2 spike (S) and nucleocapsid (N) proteins were identified following the transduction of islets *in vitro* (63). The autopsy results of 19 deceased COVID-19 patients demonstrated the presence of SARS-CoV-2 virus infiltration in beta cells in all patients. By immunohistochemistry and immunofluorescence staining of tissue from deceased COVID-19 patients, many viral antigen-positive cells were observed in the pancreatic islets and exocrine tissue (62). Specifically, pseudokinase mixed lineage kinase domain like (pMLKL)-positive cells were detected in a small percentage of pancreatic islet samples from all COVID-19 patients at autopsy. Since SARS-CoV-2 viral infiltration results in pMLKL positivity, this is likely related to the necrotic apoptotic signaling pathway (62). SARS-CoV-2 selectively infects human pancreatic islet beta cells, leading to dysregulation of insulin homeostasis, induction of apoptosis-related signaling pathways, and apoptosis.

However, there is insufficient evidence to support that hyperglycemia in COVID-19 is driven primarily by virus-induced β-cell death or dysfunction, resulting in acute or prolonged insulin deficiency. The direct cytopathic effect on beta cells remains controversial. Moreover, there are no clinical data on pancreatic involvement in patients with mild COVID-19 or evidence of widespread β-cell destruction (9).

#### 3.1.2 SARS-CoV-2 regulates the renin–angiotensin–aldosterone system to affect pancreatic β-cells

The major regulator of the renin-angiotensin system, Angiotensin-Converting Enzyme 2 (ACE2), which is found in large quantities in the epithelia of the lung and small intestine in humans, is the functional receptor for SARS-CoV-2 and acts as an anti-inflammatory enzyme (65, 66). Angiotensin-converting enzyme (ACE) converts angiotensin I (AI) to angiotensin II (AII), and ACE2 cleaves AI and AII to inactive angiotensin 1–9 and angiotensin 1–7, respectively, which have vasodilatory and antifibrotic effects. SARS-CoV binding and downregulation of ACE2 expression may explain some of the uncommon sequelae observed in patients with COVID-19, including glycemic abnormalities. The interaction between the SARS-CoV-2 surface-anchored spike (S) glycoprotein and the membrane ACE2 provides the possibility for viral entry into the host cell, which is initiated via the serine protease transmembrane serine protease 2 (TMPRSS2) for S protein initiation (67, 68). ACE2 mRNA is expressed in the majority of tissues, with particularly high levels observed in both the exocrine and endocrine pancreas. There is a correlation between ACE2 and glucose regulation. ACE2 is highly expressed in pancreatic islet cells, which serve as a point of entry for SARS-CoV-2, and pancreatic beta cells are particularly susceptible to SARS-CoV-2 infection via ACE2. SARS-CoV-2 has been demonstrated to disrupt the equilibrium of ACE/ACE2 homeostasis and RAAS activation. Additionally, the mRNAs that act on ACE2 in the pancreatic endocrine and exocrine glands affect pancreatic function (69, 70). SARS-CoV infection results in acute β-cell dysfunction, leading to acute hyperglycemia and transient T2DM. Over 50% of patients develop DM during the course of their hospitalization for SARS-CoV infection, whereas only 5% remain living with DM after three years of recovery from the viral infection (71, 72).

#### 3.1.3 Oxidative stress caused by SARS-CoV-2 infection damages beta cells

SARS-CoV-2 infection may alter mitochondrial dynamics and affect mitochondrial function at different levels, whereas mitochondria are essential for an adequate innate antiviral immune response, energy metabolism, and reactive oxygen species (ROS) production. Upon entry of SARS-CoV-2, the RNA genome is released and translated, producing structural and nonstructural proteins that interact with mitochondrial components. Subsequently, SARS-CoV-2 evades the mitochondria-mediated innate immune response and establishes an infection (73). SARS-CoV-2 has been demonstrated to induce mitochondrial damage through a number of mechanisms, including depolarization of the mitochondrial membrane, opening of the mitochondrial permeability transition pore and increased ROS release. This phenomenon is particularly evident in the mitochondrial electron transport chain, which may result in mitochondrial oxidative damage (74).

Emerging evidence indicates that COVID-19 hijacks the mitochondria of immune cells, replicates within mitochondrial structures, and disrupts mitochondrial dynamics, causing cell death (75). In addition, prolonged COVID-19 after acute infection leads to mitochondrial dysfunction, redox state imbalance, impaired energy metabolism, and chronic immune dysregulation (75). A total of 4.5% of omicron-infected individuals and 10.8% of delta variant-infected individuals experienced the debilitating syndrome of long COVID-19 (76). Proper mitochondrial function is essential for nutrient sensing and insulin secretion by beta cells. Compared with other cells, pancreatic beta cells are particularly susceptible to oxidative stress with increased DNA damage due to high ROS production and severe deficiencies in antioxidant capacity (77). These findings suggest that oxidative stress attributed to SARS-CoV-2 may play a role in β-cell failure that cannot be ignored.

### 3.2 Mechanisms by which COVID-19 affects glucose metabolism

In addition to the direct effects of the SARS-CoV-2 virus on the pancreas, the presence of inflammation, an unregulated immune response and stress also affect the host’s systemic metabolism and insulin efficacy, which in turn leads to abnormalities in glycemic levels. A variety of metabolic processes, including glycogenolysis, galactose degradation and glycolysis, are inhibited in SARS-CoV-2-infected individuals as a result of pathway enrichment analysis across tissues (75).

#### 3.2.1 Insulin resistance triggered by overactivated inflammation

COVID-19 may trigger insulin resistance, leading to chronic metabolic disorders that do not exist prior to infection. Severe SARS-CoV-2 infection and its associated hyperinflammation and cytokine activation affect insulin target tissues (primarily the liver, muscle and adipose tissue), reducing insulin sensitivity and causing hyperglycemia (33, 78). Metabolic inflammation also weakens the immune system, limiting the body’s ability to respond to infection, impeding the healing process, and lengthening the recovery period (33). Moreover, SARS-CoV-2 induces a cytokine storm, an exaggerated immune response with broad-spectrum cytokine production, which creates a systemic aggressive proinflammatory state. Elevated cytokine levels inhibit insulin signaling, leading to the development of insulin resistance and increased infiltration of adipose tissue with macrophages (79). Excessive activation of additional inflammatory variables, such as elevated neutrophil, IL-6, and CRP levels, as well as immune response abnormalities, such as decreases in lymphocyte, monocyte, CD4+, and CD8+ T-cell counts, results in insufficient insulin production and systemic insulin resistance (7). These conditions lead to impaired glucose regulation and new-onset DM. A study of the cytokine profiles of patients with acute COVID-19 and post-COVID-19 revealed that the levels of macrophage inflammatory protein-1 beta (MIP-1beta) and TNF were significantly increased in the serum of patients with COVID-19 compared with healthy controls; in the serum of patients who recovered from COVID-19, the levels of IL-1beta, IL-2, IL-4, IL-7, IL-8, IL-10, IL-13, IL-17, granulocyte colony stimulating factor (G-CSF) and IFN-γ were also increased (80, 81). Overall, inflammatory scores are increased in patients with COVID-19 and in patients recovering from COVID-19, and inflammatory scores are correlated with HOMA-IR (80). The synergistic effect between COVID-19 and T2DM may amplify the inflammatory response and further downregulate the interferon response, which may lead to greater symptomatic severity and poorer glycemic control in these patients (82).

#### 3.2.2 The integrated stress response attenuates insulin efficacy

**The** ISR is a central regulatory network of signals induced upon dysregulation of protein homeostasis and is capable of integrating various intracellular stresses, including dysregulation of protein homeostasis, nutritional deficiencies, viral infections, and redox imbalances (83). Viral infection triggers ISR, and its impact on metabolism-related diseases cannot be underestimated. The SARS-CoV-2 RNA fragment activates protein kinase R (PKR), which phosphorylates insulin receptor substrate 1 (IRS-1) serine, resulting in insulin resistance. During ISR, at least two of the four serine/threonine kinases activated by various stressors, PKR and PERK, can downregulate insulin receptor substrate serine phosphorylation via the insulin signaling pathway, reducing insulin action (78).

#### 3.2.3 Stress response

Similar to other viruses, SARS-CoV-2 infection may induce a stress response that reduces insulin production, releases counterregulatory hormones, including cortisol and adrenaline, induces excessive gluconeogenesis and impairs glucose disposal, thereby causing transient hyperglycemia (84–86). Although these mechanisms may not necessarily cause DM, they should be evaluated. In acute disease, cortisol, adrenaline and glucagon are released as a stress response to stimulate gluconeogenesis in the liver, resulting in transient hyperglycemia. Hyperglycemia induced by these processes may even lead to glucotoxicity in beta cells, which further reduces insulin secretory function (86, 87). In addition, high doses of steroids and inflammatory cytokines impair insulin secretion (88).

#### 3.2.4 Insulin resistance and reduced adiponectin levels

Obesity, metabolic syndrome, and T2DM have all been linked to dysfunctional adipokine production. Adipose tissue regulates beta cell function, mass, and insulin sensitivity in peripheral tissues (89). The lipocalin-leptin ratio is a biomarker of metabolic health and adiposity function and was found to be severely lower in the serum lipocalin group than in the ARDS-positive group. Adipsin, a hormone known to promote β-cell survival and insulin secretion, is also reduced in patients with COVID-19 (90). Therefore, it was hypothesized that COVID-19 may contribute to the development of insulin resistance and hyperglycemia by causing adipocyte dysfunction (78, 91).

#### 3.2.5 Mechanisms by which therapeutic agents for COVID-19 affect blood glucose

**Figure 1:**
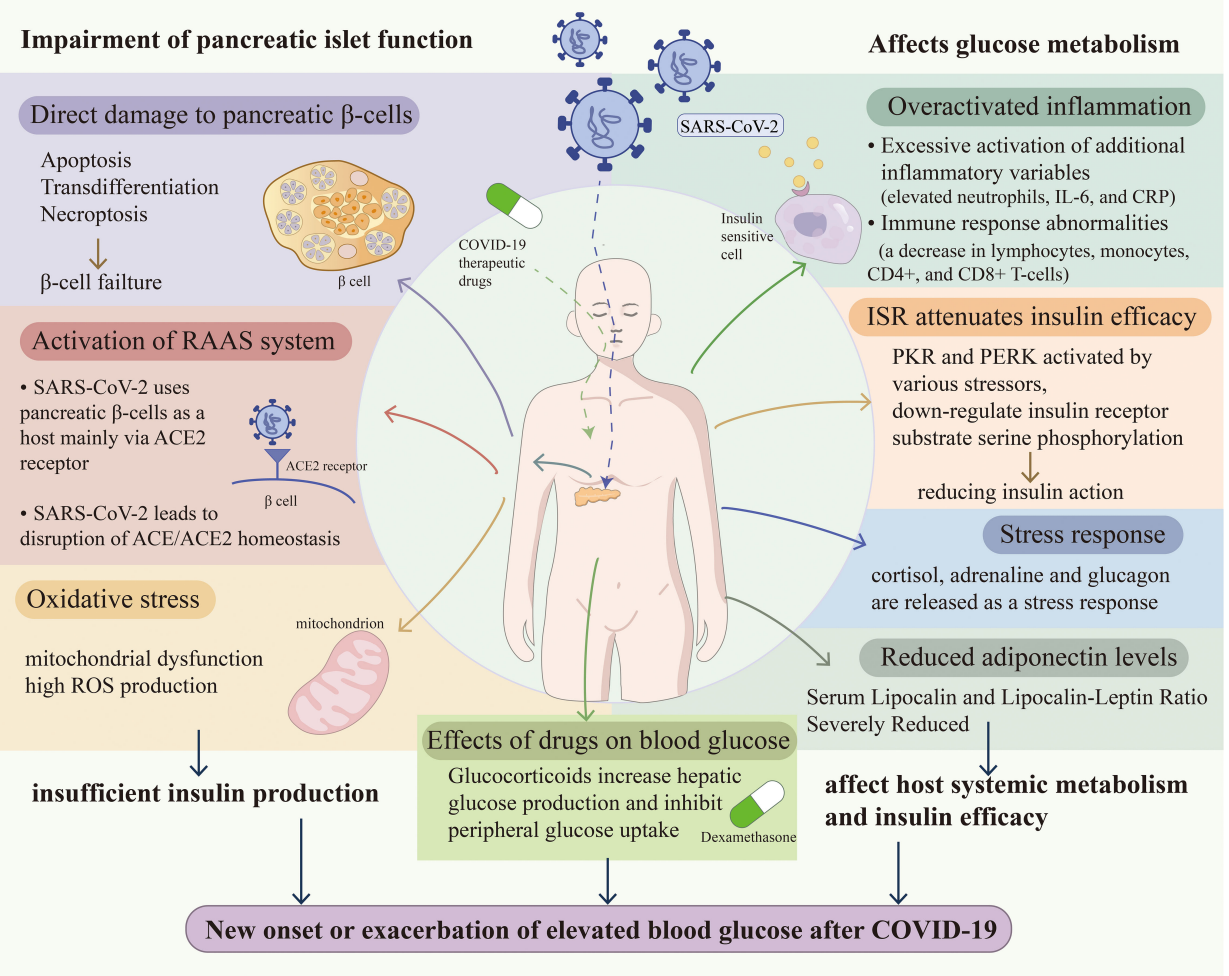
Demonstrates the mechanism by which COVID-19 contributes to poor glycemic control in new-onset patients and exacerbates DM by affecting pancreatic islet function and glucose metabolism.

Glucocorticoids have anti-inflammatory and immunomodulatory effects, while disruption of glucose metabolism when applied for long periods of time or in large amounts is a common side effect (92). Dexamethasone, a long-acting glucocorticoid, remains the most effective treatment for patients with severe SARS-CoV-2 infection requiring oxygen therapy or ventilation. However, glucocorticoids increase hepatic glucose production and inhibit peripheral glucose uptake by activating glucocorticoid receptors in multiple tissues, leading to interorgan crosstalk and inducing the expression of hepatic gluconeogenic genes (93). In addition, dexamethasone induces the apoptosis of pancreatic beta cells by upregulating the expression of the TNF-related apoptosis-inducing ligand (TRAIL) pathway and TRAIL death receptor (94). Among the 254 nondiabetic patients receiving dexamethasone for COVID-19, approximately half developed hyperglycemia (95). In particular, the risk of hyperglycemia is increased in patients receiving high doses (12–24 mg per day) of dexamethasone (96). The mechanism of action of SARS-CoV-2 in causing DM is shown in **Figure 1**.

### 3.3 Impact of COVID-19 on cardiovascular risk factors and complications of DM

#### 3.3.1 Impact of COVID-19 on cardiovascular risk factors

##### 3.3.1.1 Effect of COVID-19 on coagulation function

There are systemic dysregulations of coagulation, angiogenesis, and fibrosis in COVID-19 patients. Evidence suggests that COVID-19 infection significantly increases the likelihood of thromboembolic events, which are the leading cause of death (97, 98). The first evidence of abnormalities in coagulation parameters associated with COVID-19 infection appeared in early reports from China, as follows. The baseline characteristics of the first 99 patients hospitalized in Wuhan revealed that activated partial thromboplastin kinase time was elevated in 6% of patients, plasminogen time was elevated in 5%, and D-dimer was elevated in 36% (99). The results of another study demonstrated that patients who died from SARS-CoV-2 infection presented significantly elevated levels of D-dimer and fibrin degradation products. In this study, which included 183 Chinese patients with COVID-19 infection, 71.4% of the deaths met the criteria for diffuse intravascular coagulation, whereas only 0.6% of the survivors died (100). Notably, the incidence of venous thromboembolism is greater in patients diagnosed with SARS-CoV-2 infection (101). The long-term consequences of a fibrotic/thrombotic pancreas may also indirectly result in beta cell dysfunction, which could lead to the development of late-onset DM in COVID-19 patients (102). The mechanism behind this may be that SARS-CoV-2 infection induces endothelial injury and systemic inflammation, leading to the release of prothrombotic cytokines (e.g., IL-6, TNF-α). These cytokines upregulate tissue factor expression, activate platelets, and inhibit fibrinolysis via increased plasminogen activator inhibitor-1 (PAI-1), collectively predisposing to a hypercoagulable state (103, 104) Recent studies have found that fibrin binds to the SARS-CoV-2 spike protein, forming a pro-inflammatory clot that leads to systemic thromboinflammation in COVID-19 (105).

##### 3.3.1.2 COVID-19-related hypertension

COVID-19 elevates patients’ blood pressure, may lead to new-onset hypertension, and worsens blood pressure control in those with preexisting hypertension, which is a risk factor for its associated adverse outcomes (106, 107). A study revealed that COVID-19 patients had significantly higher systolic and diastolic blood pressures at discharge than at admission (106). In the long run, COVID-19 also increases systolic and diastolic blood pressure in nonhospitalized patients, as in a study in which more than one in six patients with new or worsening hypertension was reported at the one-year follow-up after COVID-19 rehabilitation (108). The receptor protein for SARS-CoV-2, ACE2, negatively regulates RAAS activation primarily by converting angiotensin 1 and 2 to angiotensin 1–9 and 1–7, respectively. The increase in blood pressure after COVID-19 may be due to the downregulation of ACE2 as well as the concomitant elevation of prohypertensive angiotensin 2 levels in COVID-19 patients (109). In addition, reduced physical activity, sleep disturbances, unhealthy diets, psychological stress and anxiety states, and a lack of access to healthcare during the pandemic may all be linked to worsening blood pressure regulation (110).

##### 3.3.1.3 Effects of COVID-19 on lipid metabolism

Cellular cholesterol plays a crucial role in SARS-CoV-2 entry and replication. Patients with severe COVID-19 have low levels of cholesterol, high-density lipoprotein and low-density lipoprotein. The rich lipid stores in adipocytes not only promote lipid raft formation on the cell membrane but also promote ACE2 expression, thereby facilitating viral entry. Thus, SARS-CoV-2 enables rapid adipocyte replication and expansion, leading to adipose tissue dysfunction and insulin resistance (90, 111). Metabolomic analyses suggested that patients with a history of SARS-CoV infection continued to exhibit dysregulated lipid metabolism after 12 years. The serum concentrations of free fatty acids, lysophosphatidylcholine, lysophosphatidylethanolamine, and phosphatidylglycerol are significantly greater in patients with a history of SARS-CoV infection than in individuals without a history of infection (112).

#### 3.3.2 Impact of COVID-19 on cardiovascular complications

The ACE2 receptor, which provides a direct infection pathway for SARS-CoV-2, is highly expressed in the heart. Virus-induced inflammatory cell infiltration and the production of proinflammatory cytokines (including monocyte chemoattractant protein-1, IL-1beta; IL-6; and TNF-α) may impair cardiac function; thus, patients hospitalized in the acute phase of infection may present with myocarditis-induced heart damage and elevated serum cardiac troponin and NT-proBNP levels (113, 114). SARS-CoV-2 can cause long-term effects on the cardiovascular system, with cardiovascular symptoms of long COVID-19, such as fatigue, chest pain, tachycardia, and palpitations. After the first 30 days of infection, COVID-19 patients are more susceptible to cardiovascular disease, which includes cerebrovascular disease, cardiac arrhythmias, ischemic and nonischemic heart disease, pericarditis, myocarditis, heart failure, and thromboembolic disease. The risk of cardiovascular disease in patients with SARS-CoV-2 has been shown to increase in the first 30 days of infection (115). Prolonged chronic inflammation and cellular damage prompt fibroblasts to secrete extracellular matrix molecules and collagen, leading to fibrosis. SARS-CoV-2 infection produces acute cardiac injury and pathological responses to viral myocarditis, such as endothelial damage and microthrombosis, which can lead to the development of coagulation dysfunction and increase the risk of coronary intravascular thrombosis (113). In addition, chronic hypoxia caused by COVID-19 and increased pulmonary artery pressure and ventricular strain may further accelerate the development of cardiac injury in patients with neocoronary pneumonia (109, 116).

#### 3.3.3 Effects of COVID-19 on diabetic nephropathy

The effect of ACE2 on chronic kidney disease (CKD) remains controversial. In the case of COVID-19, diabetic patients have an approximately twofold increase in the mean ACE2 mRNA level, which may increase the severity and/or risk of SARS-CoV-2 renal infection (117). In contrast, some believe that ACE2 has the potential to slow the progression of experimental diabetic chronic kidney disease. In patients with AKI, triggering the ACE2/angiotensin (1–7)/MasR axis may be nephroprotective; however, in some cases, it may accelerate kidney injury in CKD and AKI (40, 118). A comparison of the renal histopathology of COVID-19 patients and non-COVID-19 patients revealed that in the renal cortex, the proximal renal tubules exhibited significant acute tubular injury, as evidenced by the absence of the brush border and necrosis of epithelial cells in the lumen of the tubules, and microthrombi were observed in the peritubular capillaries and glomeruli (119).

#### 3.3.4 Microvascular injury due to COVID-19

**Figure 2:**
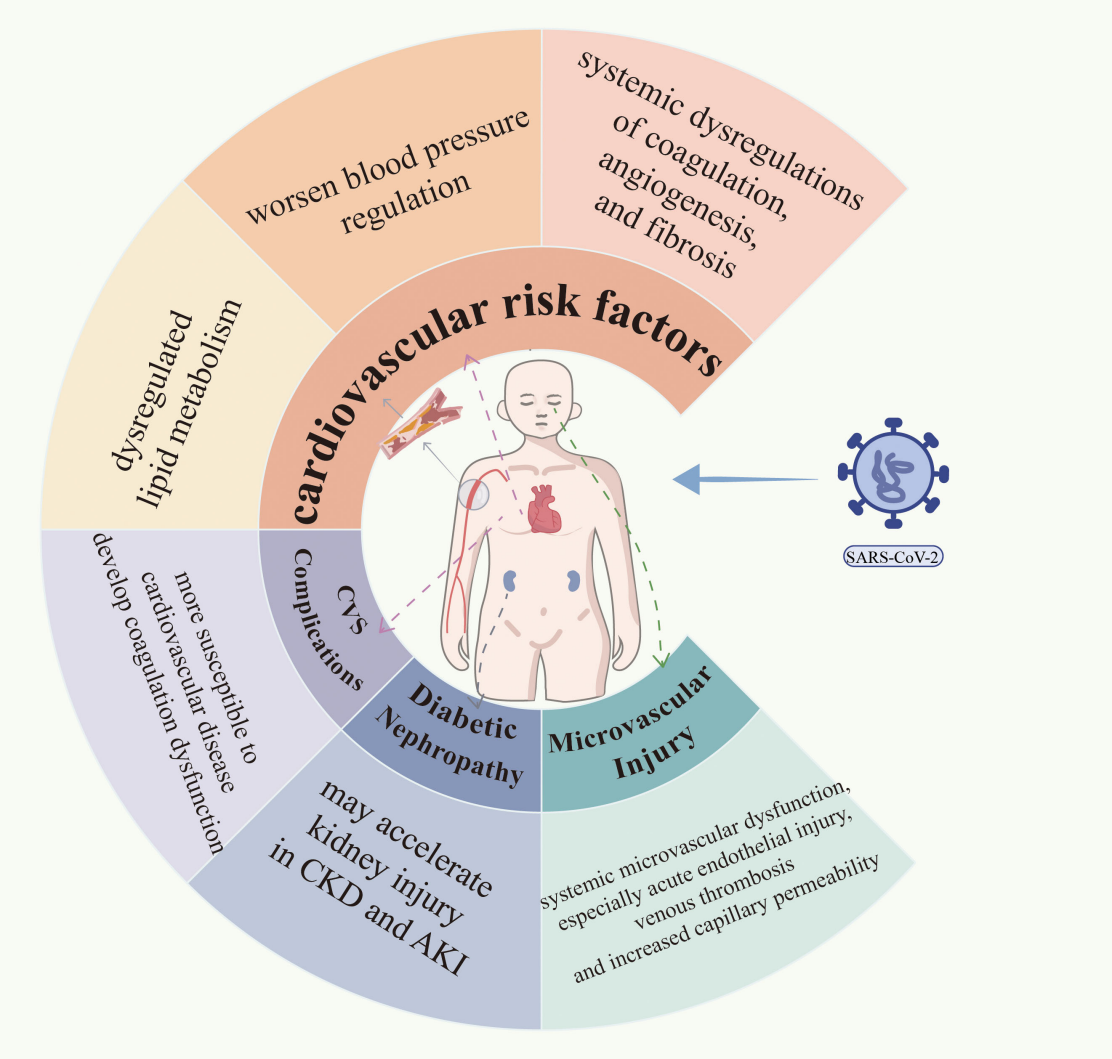
Shows the effects of COVID-19 on cardiovascular risk factors and diabetic complications.

DM-induced endothelial dysfunction is a key and initiating factor in the development of macrovascular and microvascular complications in DM, with mechanisms associated with decreased NO release, increased oxidative stress, increased production of inflammatory factors, abnormal angiogenesis and impaired endothelial repair (120). In addition to lung infection, severe COVID-19 is associated with systemic microvascular dysfunction, especially acute endothelial injury, venous thrombosis and increased capillary permeability. This is probably related to the proinflammatory cytokine storm caused by SARS-CoV-2 infection (110, 121). Owing to the severe negative effects that DM has on blood vessels at all levels throughout the body, these circulatory impairments may contribute to the development of diabetic complications, including diabetic retinopathy, neuropathy and nephropathy. Landecho et al. investigated new retinal changes in patients hospitalized for COVID-19-associated pneumonia and reported that new retinal changes were observed in 22% of patients 14 days after discharge, as assessed by fundoscopy, optical coherence tomography (OCT) and OCT angiography (122). In addition, the impact of COVID-19 on complications such as diabetic foot and diabetic retinopathy has been associated with untimely screening and insufficient resources for care in the context of epidemic control. While SARS-CoV-2-induced endothelial dysfunction and systemic inflammation may exacerbate microvascular injury, the primary drivers of diabetic microvascular complications—chronic hyperglycemia, advanced glycation end products (AGEs), and oxidative stress—remain central. COVID-19 likely amplifies existing metabolic derangements rather than directly initiating microangiopathy. The effects of COVID-19 on cardiovascular risk factors and diabetic complications are illustrated in **Figure 2**.

## 4 Conclusion

In conclusion, the global epidemic of COVID-19 has had a significant effect on the management of DM, with increased morbidity and challenges to glycemic control. The intricate interplay between SARS-CoV-2 and DM highlights the urgent need for in-depth mechanistic investigations and customized management strategies. Despite the recent observation that the symptoms of SARS-CoV-2 infection are becoming less severe, the long-term consequences of the disease, also known as long-term COVID-19, are gradually becoming more apparent and attracting attention. Furthermore, the issue of glycemic management after infection by SARS-CoV-2 continues to present a challenge for clinical practitioners. In the future, research efforts should focus on elucidating the mechanisms by which long-term COVID-19 affects blood glucose levels and the long-term impact on DM prognosis. Additionally, innovative, personalized therapeutic protocols for these patients are needed.
